# A Neonate With MuSK Congenital Myasthenic Syndrome Presenting With Refractory Respiratory Failure

**DOI:** 10.3389/fped.2020.00166

**Published:** 2020-04-16

**Authors:** Yanhua Shen, Bo Wang, Xia Zheng, Wenwen Zhang, Hailan Wu, Mingyan Hei

**Affiliations:** ^1^Neonatal Center, Beijing Children's Hospital, Capital Medical University, Beijing, China; ^2^National Center for Children's Health, Beijing, China

**Keywords:** *MUSK* gene, congenital myasthenic syndrome, respiratory failure, Chinese, neonate

## Abstract

This was a Chinese neonatal congenital myasthenic syndromes case caused by muscle skeletal receptor tyrosine kinase gene mutations, which have not been recorded in the Human Gene Mutation Database. The newborn girl had refractory respiratory failure from birth to death, and failed extubation seven times. She had two heterozygous mutations: a non-sense mutation c.2062C>T (p.Q688X) inherited from father and a missense mutation c.2324T>C (p.F775S) inherited from mother, which was predicted pathogenic and harmful by bioinformatic softwares SIFT, PolyPhen_2 and REVEL. She positively responded to Neostigmine, but her parent quitted treatment when Pyridostigmine Bromide (2 mg/kg Q12 h) had been given for 8 days. She died 2 days after she was taken home by her parents on age of 56 days.

## Background

Congenital myasthenic syndrome (CMS) is hereditary neuromuscular disorder and is a group of neuromuscular junction disorders caused by genetic defects (1, 2). Usually, CMS is diagnosed during infancy and is induced by an infection, stress, or excessive exercise, and the typical symptoms are weakness of muscles and fatigue, with possible progression to respiratory failure (3). The first gene found to be related to CMS was the cholinergic receptor nicotinic epsilon subunit (CHRNE) gene, which was reported by Ohno et al. (4). To date, more than 30 genes have been found to be related to this disease (5–7). Approximately 50% of CMS cases were caused by mutations in all subunits of AChR (2). About 15–20% of cases were caused by a mutation of RAPSN gene, an acetylcholine receptor-associated synaptic protein-encoding gene (8). CMS caused by muscle skeletal receptor tyrosine kinase gene (*MUSK*) mutation is rare, which encodes a muscle-specific kinase (9). *MUSK* mutation was first reported in 2004 (9). To date, 23 CMS cases worldwide have been reported to be related to *MUSK* mutation, among them 17 cases were non-neonates (2, 3, 10, 11), six cases were neonates who were from France (9), the United States (12), Italy (13), Turkey (14), and Germany (15), respectively. Among the 23 MuSK CMS cases ever reported, the typical clinical manifestation was the mild muscle weakness of the eyes and limbs (16), and the only Chinese patient with *MUSK* mutation CMS was a man who started to have symptom of limb girdle when he was 8 years old and was diagnosed when he was 30 years old (3). Herein, we report the case of a female neonate with CMS caused by *MUSK* mutations which, to the best of our knowledge, have never been reported in a Chinese neonate.

## Case Presentation

The patient was the second baby of the family. She was a girl born in a local hospital by spontaneous vaginal delivery at the gestational age of 37+6 weeks with a birth weight of 2,850 g (at the 50th percentile) and Apgar scores of 9 at 1 min, 6 at 5 min, and 8 at 10 min after birth. There was no abnormal maternal history during this pregnancy. Her parents denied any abnormal family history. She had a 7-year-old brother who was normal. She started to have progressive cyanosis, grunting, and tachypnea soon after birth and was intubated in the delivery room. Chest X-ray did not find significant abnormalities, but systemic broad-spectrum antibiotics were started after blood culture was taken and 1 dose of pulmonary surfactant was given at 2 h after birth. Feeds with formula via nasal gastric (NG) tube feeding were started when she was 7 days old. The infant did not show positive response, and she failed extubation for three times in the local hospital (when she was 4, 7, 18 days old, respectively). She was transferred on ventilation to Beijing Children's Hospital (BCH) when she was 23 days old.

**Figure 1:**
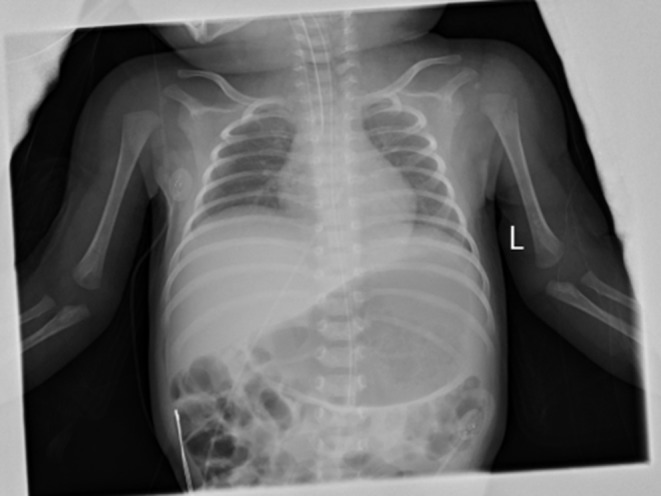
Anterio-posterial Chest and abdominal X-ray on DOL 23. The anterio-posterial chest and abdominal X-ray on DOL23 showed a slightly decreased lung volume with normal lung parenchyma and heart image. The right diaphragm was at the right 6–7 intercostal space and was 1 intercostal space higher than the left side.

During the hospitalization in BCH, her vital signs were stable, but she was generally inactive. Her muscle tone was slightly decreased. Primary reflexes of sucking, rooting, and grasping could not be induced. The Moro reflex was symmetrical but incomplete. Otherwise her physical examination found no abnormalities. NG tube feeding with formula (140 ml·kg-1·day-1) was well-tolerated. A systemic antibiotic was administered for 48 h till a negative culture was obtained. The infant was stooling well, and the urine output was normal. All lab investigations, including the cerebrospinal fluid investigations, of the infant were normal. The blood culture, blood and urine metabolic screening were negative. Her chest X-ray image showed normal lung parenchyma but a slightly decreased lung volume (with the right diaphragm at the 6th−7th intercostal space, and the left diaphragm at the 7th−8th intercostal space) (Figure 1). The bilateral diaphragmatic movement under ultrasound was reduced. None of the cranial CT or MRI, ECG or echocardiography, aEEG or vEEG, laryngoscopy, or fiberoptic bronchoscopy examinations found any abnormalities. The parents refused the neuroelectromyogram examination for the baby girl as according to their words “did not want to take this traumatic examination for our daughter.”

She failed four times of extubation after being admitted to BCH, requiring persistent synchronized intermittent mandatory ventilation (SIMV) support. The SIMV settings were low (Table 1) and blood gases were normal. A Neostigmine test was carried out on the infant when she was 45 days old. In detail: The Neostigmine was administered at a dose of 0.04 mg·kg-1 by intramuscular injection (15, 17). She responded quite positively to Neostigmine. At 20 min after the injection, her rate of breathing had decreased from 60–70·min-1 to 50·min-1 and the heart rate had decreased from 200–205·min-1 to 175–180·min-1. At 35 min after the injection, her rate of breathing was 35·min-1 and her heart rate was 135–150·min-1. After the Neostigmine test, an oral pyridostigmine bromide was given at a dose of 2 mg·kg-1 Q12 h per day. The vital signs of the infant were stable on biphasic CPAP (settings: PIP 9cmH_2_O, PEEP cmH_2_O, Rate 30 min-1, FiO_2_ 25%). The enteral feeding (140 ml·kg-1·day-1) by NG tube was well-tolerated. No re-intubation was required. When she was 54 days old, her parents quitted any further treatment and took her home because they wanted a normal healthy daughter but they could not get absolute guarantee from physicians. The infant died at home on when she was 56 days old from respiratory failure.

**Table 1:** Pre-extubation settings of SIMV ventilation in BCH.

**Age on extubation d**	**PIP cmH_**2**_O**	**PEEP cmH_**2**_O**	**Rate ·min^**−1**^**	**Flow liter·min^**−1**^**	**Ti s**	**FiO2 %**	**Notes for the attempt**
28	14	5.5	40	8	0.45	21	Failed
33	14	5	35	8	0.4	21	Failed
40	13	5	35	8	0.4	21	Failed
43	13	6	40	8	0.4	25	Failed
45^*^	13	6	35	8	0.4	23	Successful^*^

* *The age and the results on the day when Neostigmine was administered*.

*BCH, Beijing Children's Hospital; FiO_2_, fraction of inspired oxygen; PIP, peak inspiratory pressure; PEEP, positive end expiratory pressure; SIMV, synchronized intermittent mandatory ventilation; Ti, inspiratory time*.

**Figure 2:**
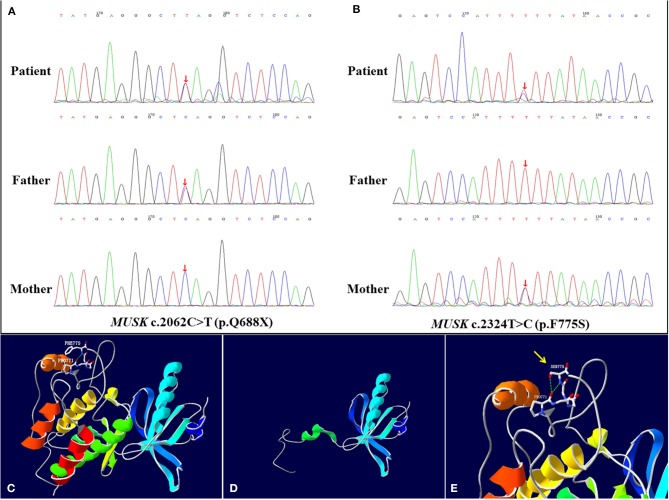
*MUSK* mutation analysis and models of the predicted protein structure of MuSK. **(A,B)** show the *MUSK* mutations in the patient and her parents, as confirmed by Sanger sequencing. The patient harbored compound heterozygous mutations c.2062C>T (p.Q688X) and c.2324T>C (p.F775S) of the *MUSK* at exon 14. **(A)** In the patient, one allele bears the non-sense mutation c.2062C>T (p.Q688X), which was inherited from her father. **(B)** The other allele bears the c.2324T>C (p.F775S) missense mutation, which was inherited from her mother. **(C–E)** are the re-constructed three-dimensional (3D) structure of normal MuSK and the predicted MuSK protein structures caused by the 2 mutations. **(C)** Total view of the 3D structure of normal MuSK. The green dotted line indicates hydrogen bonds between groups. The Phe-775 forms a hydrogen bond with the backbone atoms of Pro-771. **(D)** The mutation c.2062C>T (p.Q688X) results in a truncation of the protein and disruption of the protein kinase domain structure. **(E)** The 3D model of the MuSK amino acid substitution of p.F775S (c.2324T>C), from phenylalanine to serine, highlighted by the yellow arrow. The two hydrogen bonds interacting with Pro-771 are formed when Phe-775 is mutated to Ser, which is different from the wild type.

The result of gene mutation analysis of the infant and her parents by targeted whole-exome next-generation sequencing showed that the infant harbored two novel heterozygous mutations in the *MUSK*, and were subsequently confirmed by Sanger sequencing (Figures 2A,B). A novel heterozygous non-sense variant, c.2062C>T (p.Q688X), was detected in exon 14 of the *MUSK* and was rated to be likely pathogenic according to the American College of Medical Genetics and Genomics guidelines. This might cause the loss of protein function. This mutation was also detected in her father but not in her mother, indicating that it was paternally inherited. Another mutation, c.2324T>C, which resulted in a change of amino acid p.F775S, was a missense mutation. Three bioinformatics prediction software (SIFT, PolyPhen_2 and REVEL) predicted the c.2324T>C mutation to be harmful. After searching the mutation records of normal population in 1000 Genome Project, NHLBI Exome Sequencing Project (ESP6500), Exome Aggregation Consortium (ExAC_ALL), Exome Aggregation Consortium East Asian (ExAC_EAS), no information of the frequency of the two mutations in normal population were found. Sanger sequencing was also performed on the DNA of her parents, which revealed that her mother was heterozygous for the c.2324T>C variant whereas her father did not carry it. The SWISS-MODEL workspace (http://swissmodel.expasy.org) was used to characterize the effect of the mutations on the protein of MuSK. We used the crystal structure of the cytoplasmic domain of unphosphorylated MuSK of rat (PDB# 1LUF) with a 97.09% sequence identity of human MuSK for simulation. We found that the structures of the protein resulting from these two mutations were predicted and compared with that of the wild type, whereupon significant structural differences were observed (Figures 2C–E). The predicted structure of the non-sense mutation c.2062C>T (p.Q688X) revealed a significant structural loss of the protein that might cause the loss of protein function. The missense mutation c.2324T>C occurred in a domain of protein which was conserved in different species. The two mutations were located in the protein kinase domain of the scaled linear model of the *MUSK*. The transmembrane domain includes amino acids 496–516.

## Discussion

*MUSK* is located on chromosome 9 at 9q31.3q.32 and encodes a muscle-specific kinase (9). This kinase is a transmembrane protein that is associated with the acetylcholine receptor, which is the primary component of major synaptic skeletons during the establishment of postsynaptic membranes in skeletal muscle development (2). This baby girl had two heterozygous mutations in the *MUSK* inherited from her father and mother, presenting refractory respiratory failure from right after birth to her death. The clinical manifestations of this baby girl were similar to those of 6 previously reported cases diagnosed, among whom some were diagnosed within the neonatal period (2, 3, 10).

The refractory respiratory failure of the patient could not be explained by any other reasons, for there was no disorder found in the respiratory tract, cardiopulmonary system, and central nervous system, and in her brain function. The c.2324T>C (p.F775S) *MUSK* mutation has not been reported in the literature, and it was predicted by SIFT, PolyPhen_2, and REVEL bioinformatics softwares to be harmful, indicating its pathogenic significance. This was consistent with the patient's clinical manifestations, disease course, and final fatal outcome. Variant c.2062C>T (p.Q688X) is non-sense but variant c.2324T>C (p.F775S) is in the tyrosine kinase region of the MuSK. It was hypothesized that mutations in the protein kinase region may be specific for late-onset limb-girdle weakness (16). But in our case, the infant did not show any signs of limb weakness during her hospitalization period. We might not have had enough time to assess whether the infant was going to show signs of late-onset limb-girdle weakness or not, because her parents quitted further treatment and took her home. Among the previously reported 6 neonatal *MUSK* CMS cases, 2 of them had c.2368 G>A mutation (9, 13), while the *MUSK* mutation sites of the other 4 patients were different from each other. The 2 mutations found in our patient were different from the other 6 neonates, had no information about the frequency in normal population, and were not reported yet.

CMS is a rare hereditary disease caused by a dysfunction of neuromuscular transmission. Result of an electrophysiology investigation is a very important clinical evidence for the diagnosis confirmation. Unfortunately, we did not have the results of the neuroelectromyogram examination as per her parents' request, which we fully understood. The common symptoms Of CMS are weakness of the eye muscles, extraocular muscles, and limb muscles (3). The clues for considering a CMS diagnosis for this patient were (1) her shallow breathing and decreased lung volume on chest X-ray, indicating weakness of the breathing muscles; and (2) the negative results of laboratory examinations, the normal imaging results of the airway and lungs, brain, heart, and abdomen, and the normal results from investigations of her brain, liver, and renal functions. Although neuroelectrophysiological examination is of great significance for the diagnosis of CMS (15), it is difficult to implement in small infants or newborns owing to its invasive nature. The test with Neostigmine, a cholinesterase inhibitor, is more often used clinically for the diagnosis of myasthenia gravis (18, 19) or CMS (17). The positive or negative result of this test is determined through close assessment of the patient's response to Neostigmine before and at every 10 min after the injection. In this case, the respiratory failure was significantly improved after Neostigmine injection, requiring no further re-intubation, which was apparently different from what happened before. Regrettably, the parents did not continue the treatment and took the infant home, where she died of respiratory failure 2 days later. This outcome implies that there was no way to estimate the real prognosis of this patient had she been continued on oral Neostigmine intervention. There is a lack of literature support for positive respond of acetyl cholinesterase inhibitors to *MUSK* CMS, and there are even reports of worsening with these medications (11). The positive therapeutic response of this infant to Neostigmine was interesting, which we could only explain this by the possible unique characteristics of neonates and different pharmacokinetic response of neonates comparing to adults. For this infant, it was still not sure for how long time and to what extent Neostigmine might function well for this baby because her parents quitted further treatment, took the baby home and she died soon. It was reported that immunotherapy has been tried for CMS patients (16, 20) but no positive effect has been shown, we did not try any immunotherapy for this MUSK CMS patient.

In recent years, whole-exon sequencing, which can read approximately 75% of an exon with more than 20X coverage (21), has been frequently used to study CMS gene mutations. Any putative mutation should be further confirmed by Sanger sequencing (22), as performed for the 2 heterozygous mutations found in this patient. Both the *MUSK* c.2324T>C (p.F775S) variant and c.2062C>T (p.Q688X) variant were concluded to be novel mutations.

## Data Availability Statement

The raw data supporting the conclusions of this article will be made available by the authors, without undue reservation, to any qualified researcher.

## Ethics Statement

The studies involving human participants were reviewed and approved by Beijing Children's Hospital. Written informed consent to participate in this study was provided by the participants' legal guardian/next of kin.

## Author Contributions

YS and BW contributed to the conception of the work, the acquisition, analysis and interpretation of data for the work, and drafted the work. XZ, WZ, and HW acquired, analyzed the data, and drafted the work. MH contributed to the conception and design of the work, and revised it critically for important intellectual content. All authors approved publication of the content and agreed to be accountable for all aspects of the work in ensuring that questions related to the accuracy and integrity of any part of the work are appropriately investigated and resolved.

### Conflict of Interest

The authors declare that the research was conducted in the absence of any commercial or financial relationships that could be construed as a potential conflict of interest.
